# Proactive, short-term PCSK9 inhibition after PCI in patients with coronary artery disease at high residual risk: rationale and design of the randomized HANYANG-PICK trial

**DOI:** 10.3389/fcvm.2026.1761093

**Published:** 2026-03-02

**Authors:** Woohyeun Kim, Soojung Park, Jeong-Hun Shin, Hyungdon Kook, Young-Hyo Lim

**Affiliations:** 1 Division of Cardiology, Department of Internal Medicine, College of Medicine, Hanyang University, Seoul, Republic of Korea; 2 Division of Cardiology, Department of Internal Medicine, Hanyang University Seoul Hospital, Seoul, Republic of Korea; 3 Division of Cardiology, Department of Internal Medicine, Hanyang University College of Medicine, Hanyang University Guri Hospital, Guri, Republic of Korea

**Keywords:** evolocumab, PCSK9 inhibitor, intravascular imaging, NIRS-IVUS, lipid core burden index, percutaneous coronary intervention, residual cardiovascular risk, stent failure

## Abstract

**Background:**

Despite advances in stent design and PCI optimization, stent failure remains clinically relevant in patients with coronary artery disease. This process is primarily driven by vascular injury and maladaptive healing, leading to neointimal hyperplasia, neoatherosclerosis, and recurrent ischemic events. A subset of patients remains vulnerable despite angiographically successful PCI, reflecting residual risk not fully captured by procedural assessment alone. Novel strategies to reduce this residual risk are therefore warranted. Proprotein convertase subtilisin/kexin type 9 (PCSK9) inhibitors, beyond potent LDL-C reduction, have demonstrated plaque-stabilizing effects. Preclinical data suggest that PCSK9 promotes proinflammatory cytokine release, vascular smooth muscle cell proliferation, and impaired endothelial repair—mechanisms implicated in adverse vascular responses after PCI.

**Aim:**

To determine whether proactive, short-term evolocumab improves outcomes in patients with coronary artery disease at high residual risk after PCI.

**Methods:**

The HANYANG-PICK trial is a prospective, randomized, open-label, multicenter study enrolling patients with post-PCI _max_LCBI_4mm_ ≥200 on NIRS-IVUS. Participants will be randomized 1:1 to standard care or standard care plus evolocumab 140 mg subcutaneously within 24 h of PCI and at 2 weeks. The primary endpoint is the composite of all-cause death, myocardial infarction, stroke, or any clinically driven revascularization at 12 months.

**Ethics and dissemination:**

The protocol has been approved by the Institutional Review Board of all participating centers. Written informed consent will be obtained from all participants. Results will be disseminated in peer-reviewed journals and at scientific conferences.

**Conclusions:**

The HANYANG-PICK trial is an exploratory, hypothesis-generating randomized study designed to test whether proactive, short-term PCSK9 inhibition initiated during the early post-PCI period can reduce adverse outcomes in patients with high residual risk identified by post-PCI intravascular imaging.

**Clinical Trial Registration:**

https://clinicaltrials.gov/study/NCT07084259, Identifier NCT07084259.

## Introduction

Despite advances in stent technology, intravascular imaging, and secondary prevention, stent failure still occurs at an annual incidence of approximately 1%–2% in patients with coronary artery disease undergoing percutaneous coronary intervention (PCI) (1). This entity encompasses stent thrombosis, intimal hyperplasia, and neoatherosclerosis—pathophysiological processes driven by procedure-related vascular injury and maladaptive healing that follow balloon angioplasty and stent deployment (1–3). Notably, a subset of patients continues to experience these complications despite angiographically successful PCI, underscoring the presence of residual biological risk that is not fully captured by procedural or angiographic assessment alone. Identifying such individuals and developing strategies that target these biological pathways therefore remain important unmet clinical needs.

Proprotein convertase subtilisin/kexin type 9 (PCSK9) inhibitors, through potent LDL-C reduction, have been shown to improve clinical outcomes in patients with atherosclerotic cardiovascular disease (4–6). Beyond lipid lowering, these agents have been associated with favorable changes in plaque composition, including reductions in plaque volume and lipid burden, as well as thickening of the fibrous cap—features consistent with stabilization of vulnerable plaque (7–9). Preclinical evidence further indicates that PCSK9 is involved in proinflammatory cytokine release (10–12), vascular smooth muscle cell proliferation (13, 14), and impaired endothelial repair (15, 16), all of which may contribute to maladaptive vascular healing following PCI. Collectively, these observations suggest that PCSK9 inhibition may favorably influence vascular responses during the early post-PCI period (17–19).

We therefore hypothesize that proactive PCSK9 inhibition during the early vulnerable phase after PCI may attenuate adverse vascular responses associated with maladaptive healing and reduce subsequent cardiovascular risk in selected high-risk patients. This early post-PCI period encompasses both the acute inflammatory and subacute proliferative phases (20–23), during which plaque-related biological activity remains dynamic. To capture this biologically critical window, we selected a short-term, 4-week regimen of evolocumab, which provides sustained PCSK9 suppression with only one or two injections, thereby optimizing both adherence and feasibility within an exploratory, hypothesis-generating trial framework.

The HANYANG-PICK trial (HArnessing Near-infrared spectroscopy–intravascular ultrasound imaging for Yielding AdvaNced Guidance with a PCSK9 inhibitor for Improving Cardiovascular outcomes in high-risK patients) was designed to test this hypothesis in patients with high residual intraplaque lipid burden after PCI, defined as post-PCI _max_LCBI_4mm_ ≥ 200 on NIRS-IVUS. By using post-PCI intravascular imaging rather than angiographic or baseline lesion characteristics alone, this imaging-based risk-enrichment strategy aims to identify patients with persistently high residual risk despite technically successful revascularization. This prospective, investigator-initiated, randomized, open-label trial evaluates whether a proactive, short-term, 4-week course of evolocumab initiated immediately after PCI can favorably influence clinical outcomes by targeting a high-risk patient population during the early post-PCI period.

## Methods

### Study design and population

**Figure 1:**
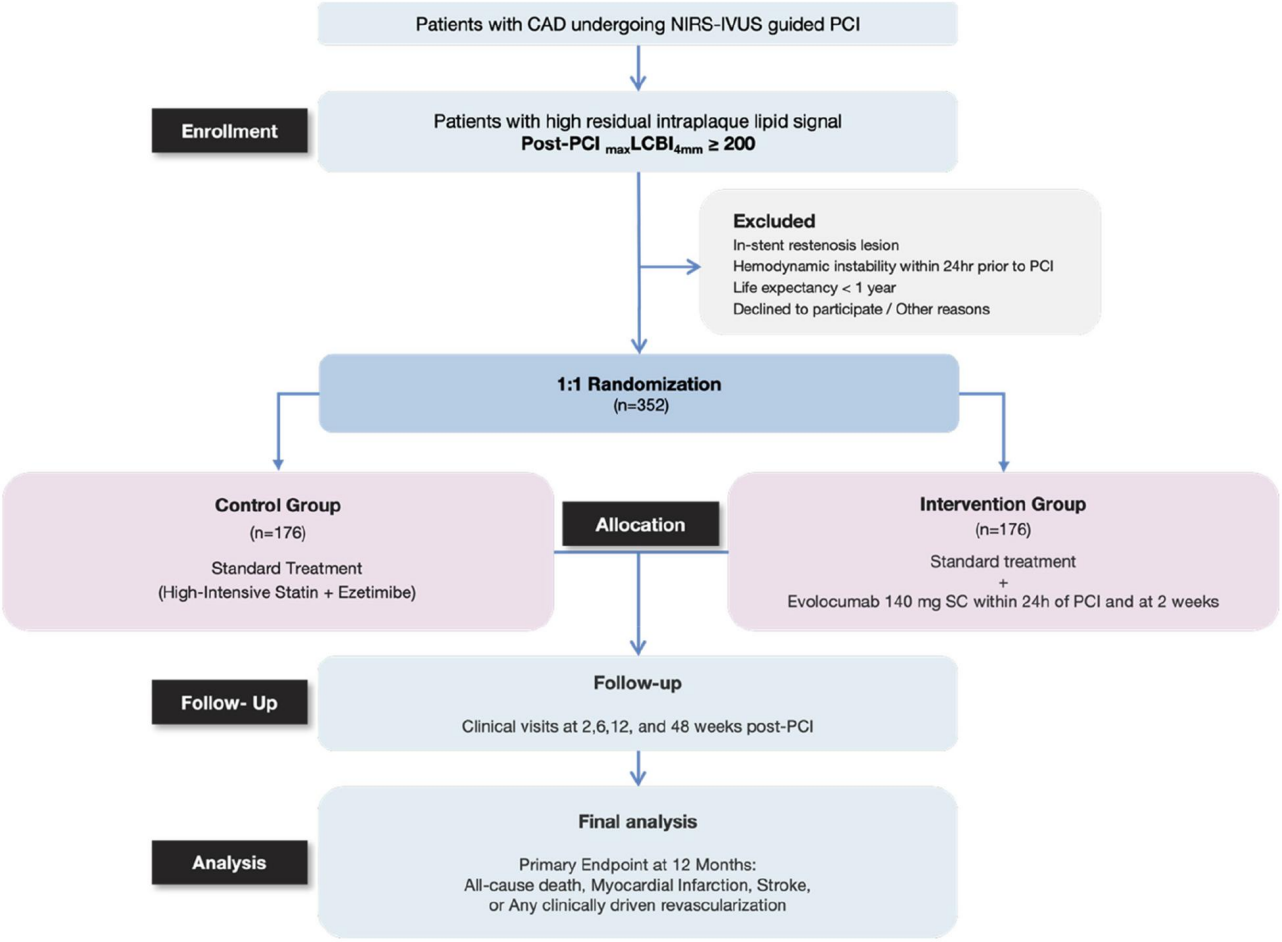
Study flow and schematic overview of the HANYANG-PICK trial design. This figure illustrates the overall study flow and design of the HANYANG-PICK trial. Patients with coronary artery disease undergoing NIRS-IVUS–guided PCI are screened immediately after PCI, and those with high residual intraplaque lipid signal defined as post-PCI _max_LCBI_4mm_ ≥200 are enrolled. Eligible patients are randomized in a 1:1 ratio to standard guideline-directed lipid-lowering therapy alone or standard therapy plus short-term evolocumab initiated within 24 h after PCI and repeated at 2 weeks. Clinical follow-up visits are scheduled at 2, 6, 12, and 48 weeks after PCI. The primary endpoint, a composite of all-cause death, myocardial infarction, stroke, or any clinically driven revascularization, is assessed at 12 months.

The HANYANG-PICK trial is a prospective, investigator-initiated, randomized, open-label, multicenter study conducted at university hospitals in Korea and the overall study design is illustrated in Figure 1. Eligible patients are aged ≥19 years with coronary artery disease, including stable angina, silent ischemia, or acute coronary syndromes, undergoing PCI with NIRS-IVUS imaging. Post-PCI _max_LCBI_4mm_ ≥200 is required for randomization. This threshold was selected to identify patients with substantial residual risk after angiographically successful PCI, as previously associated with increased adverse cardiovascular events (24, 25). Key exclusion criteria include target lesions involving in-stent restenosis (ISR), hemodynamic instability within 24 h prior to PCI, and life expectancy <1 year.

### Randomization and intervention

After confirming eligibility and obtaining informed consent, patients will be randomized 1:1 to the intervention or control arm. The intervention group will receive standard post-PCI care plus evolocumab 140 mg subcutaneously within 24 h of PCI and again at 2 weeks; the control group will receive standard post-PCI care alone, including high-intensity statin therapy. All patients are required to receive maximally tolerated high-intensity statin therapy, with ezetimibe added according to contemporary guideline recommendations, unless contraindicated.

### Endpoints

The primary endpoint is the composite of all-cause death, myocardial infarction, stroke, or any clinically driven revascularization at 12 months. Clinically driven revascularization is defined as repeat revascularization performed in the presence of recurrent ischemic symptoms and/or objective evidence of myocardial ischemia, including ischemic electrocardiographic changes, stress testing, or imaging findings, according to prespecified criteria. Secondary endpoints include each individual component of the primary endpoint and target lesion failure, defined as cardiac death, target vessel myocardial infarction, or clinically driven target lesion revascularization at 12 months.

### Sample size

In a previous observational cohort (25), patients with Post-PCI _max_LCBI_4mm_ ≥200 had a markedly higher 12-month incidence of major adverse cardiovascular events (MACE) compared with those with Post-PCI _max_LCBI_4mm_ <200 (15.1% vs. 2.2%), with the overall event rate in the unselected cohort being 7.5%. Given these findings, we hypothesized that proactive, short-term PCSK9 inhibition initiated immediately after PCI in patients with post-PCI _max_LCBI_4mm_ ≥200 could reduce their MACE rate toward the average level observed in the overall population (∼7.5%). Under this assumption, a total of 316 patients (158 per group) would provide 80% power to detect this difference at a two-sided *α* of 0.20, which was chosen to support signal detection and effect-size estimation in this exploratory, hypothesis-generating randomized trial. To account for an anticipated dropout rate of 10%, the target enrollment was set at 352 patients.

### Follow-Up and data collection

Patients will be followed at 2-, 6-, 12-, and 48-weeks post-PCI. At the 6-week visit, lipid profiles will be reassessed, and subsequent lipid-lowering therapy will be optimized in accordance with contemporary guideline-directed medical therapy targeting an LDL-C level <55 mg/dL; continuation or initiation of PCSK9 inhibitors is recommended when clinically indicated. At each visit, primary and secondary endpoints will be assessed and recorded. If a patient does not attend a scheduled visit, the investigator or research coordinator will promptly contact the patient by telephone to reschedule the appointment, thereby minimizing the risk of underreporting clinical events. All clinical endpoints will be adjudicated by an independent committee blinded to treatment allocation. Data will be recorded in a secure, password-protected electronic case report form.

### Statistical analysis

Primary analyses will use the intention-to-treat population. Kaplan–Meier survival curves will be generated for time-to-event endpoints, with between-group differences assessed by log-rank test. Hazard ratios with 95% confidence intervals will be estimated using Cox proportional hazards models. Continuous variables will be compared using Student's t-test or Mann–Whitney U test, and categorical variables with chi-square or Fisher's exact test, as appropriate. Prespecified exploratory subgroup analyses, including analyses according to sex, will be performed to assess potential effect modification.

### Ethics and dissemination

The protocol has been approved by the Institutional Review Board of all participating centers. Written informed consent will be obtained from all participants. Results will be disseminated in peer-reviewed journals and at scientific conferences.

### Trial Status

The first patient was enrolled on July 23, 2025. Patient recruitment is planned to continue for approximately 3 years, with enrollment expected to be completed by July 2028. Final follow-up is scheduled to be completed 4 years after trial initiation.

### Study governance and oversight

The HANYANG-PICK trial is an investigator-initiated study supported by DOTTER Inc. The trial will be led by the Study Chairman (Prof. Woohyeun Kim) in collaboration with the steering committee, which is responsible for the medical, scientific, and operational conduct of the study. The steering committee will oversee protocol adherence, site coordination, and data quality. An independent clinical endpoint committee, whose members are not involved in the conduct of the trial, will adjudicate all clinical events during follow-up according to prespecified definitions, using source documentation and other relevant medical records.The authors will be solely responsible for the study design, conduct, statistical analyses, and preparation of the manuscript, including drafting, critical revision, and final approval of the content. The trial will be conducted in accordance with the principles of the Declaration of Helsinki, the International Conference on Harmonization–Good Clinical Practice guidelines, and applicable regulatory requirements. Hanyang University Seoul Hospital will serve as the trial sponsor, with delegated responsibilities carried out by its Clinical Research Center to ensure compliance with national regulations governing clinical trials.

## Discussion

### Study objectives

The HANYANG-PICK trial addresses an unmet clinical need by focusing on patients with high residual risk after PCI—a population that remains vulnerable to recurrent cardiovascular events despite successful revascularization and contemporary secondary prevention strategies. The primary objective is to determine whether proactive, short-term PCSK9 inhibition, initiated during the early post-PCI period, can favorably influence post-PCI vascular responses and reduce adverse cardiovascular outcomes in this high-risk subset.

### Identifying patients with high residual risk after PCI

**Figure 2:**
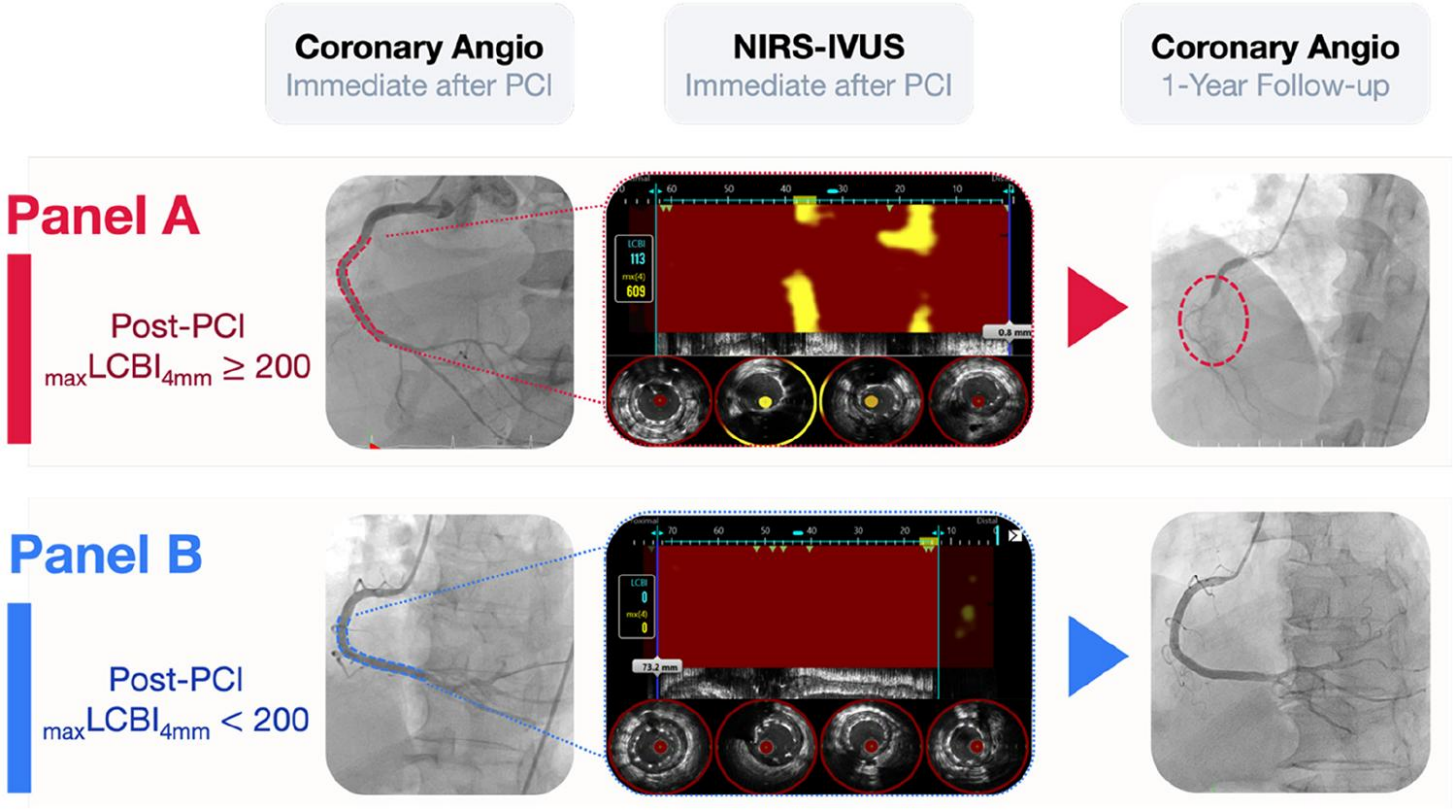
Angiographically successful PCI with divergent post-PCI _max_LCBI_4mm_ assessed by NIRS-IVUS. **(A)** Shows a representative case with post-PCI _max_LCBI_4mm_ ≥200, in which a high residual intraplaque lipid signal was detected by NIRS-IVUS despite angiographically successful PCI, followed by target lesion failure during follow-up. **(B)** Shows a representative case with post-PCI _max_LCBI_4mm_ < 200, with minimal residual lipid signal and no target lesion failure. Chemograms and corresponding IVUS cross-sectional images reveal residual intraplaque lipid burden that is not apparent on angiography.

Accurate identification of high residual risk is essential to target therapies effectively. In this trial, high residual risk is defined by the presence of residual lipid burden with a post-PCI _max_LCBI_4mm_ ≥200 as measured by NIRS-IVUS (Figure 2) (24, 25). This imaging phenotype has been reported to be associated with future adverse events, even in the setting of optimal PCI and medical therapy in previous studies (24, 25). Leveraging NIRS-IVUS not only allows for precise plaque characterization but also enables an imaging-based risk-enrichment strategy, thereby enhancing the likelihood of detecting a meaningful treatment effect.

### Mechanistic and temporal rationale for early short-duration PCSK9 inhibition

PCSK9 inhibitors have consistently demonstrated potent LDL-C lowering and significant reductions in cardiovascular events among patients who fail to achieve target LDL-C levels despite maximally tolerated statin plus ezetimibe therapy (4–6). Beyond lipid lowering, these agents have been shown to reduce plaque volume, decrease lipid burden, and increase fibrous cap thickness, thereby stabilizing vulnerable plaques (7–9). Preclinical evidence further suggests that PCSK9 may be involved in maladaptive vascular responses through promoting proinflammatory cytokine release (10–12), vascular smooth muscle cell proliferation (13, 14), and impaired endothelial repair (15, 16), processes that are implicated in the development of stent thrombosis, intimal hyperplasia, and neoatherosclerosis. Although these pathways are not directly interrogated in the present trial, early PCSK9 inhibition during the acute inflammatory and subacute proliferative phases following PCI may attenuate adverse vascular responses during this vulnerable period (17–19). A 4-week treatment course was chosen to target this biologically dynamic phase of vascular healing (20–22) while maintaining treatment feasibility, adherence, and cost-effectiveness. Importantly, the HANYANG-PICK trial is designed to evaluate the effect of proactive, short-term PCSK9 inhibition in patients with high residual risk after PCI, as defined by post-PCI intravascular imaging rather than baseline lipid levels alone.

### Limitations

The strengths of the HANYANG-PICK trial include the use of NIRS-IVUS to identify patients with high residual risk, a multicenter randomized design, and clinically relevant hard endpoints. The pragmatic 4-week treatment duration balances biological plausibility, feasibility, and considerations of adherence and cost.

Several limitations should be acknowledged. The open-label design could introduce bias; however, the use of an independent clinical endpoint committee blinded to treatment allocation is expected to mitigate this concern. The relatively short treatment duration and modest sample size may limit the statistical power to detect differences in individual components of the composite endpoint. Accordingly, this trial is positioned as an exploratory, hypothesis-generating study intended to inform the design of future confirmatory trials rather than to provide definitive efficacy estimates. In addition, although exploratory subgroup analyses according to sex are planned, the trial is not specifically powered to detect sex-specific treatment effects, and any such findings should therefore be interpreted with caution.

## Conclusion

If successful, the HANYANG-PICK trial will provide the first randomized evidence on whether proactive, short-term PCSK9 inhibition during the early vulnerable period after PCI can favorably influence clinical outcomes in patients with high residual risk identified by post-PCI intravascular imaging, potentially establishing a novel framework for imaging-guided residual risk management after coronary intervention.

## References

[1] Giustino G Colombo A Camaj A Yasumura K Mehran R Stone GW Coronary in-stent restenosis: jACC state-of-the-art review. J Am Coll Cardiol. (2022) 80(4):348–72. 10.1016/j.jacc.2022.05.017 35863852

[2] Park S-J Kang S-J Virmani R Nakano M Ueda Y. In-Stent neoatherosclerosis. JACC. (2012) 59(23):2051–7. 10.1016/j.jacc.2011.10.909 22651862

[3] Rogers C Tseng DY Squire JC Edelman ER. Balloon-Artery interactions during stent placement. Circ Res. (1999) 84(4):378–83. 10.1161/01.RES.84.4.378 10066671

[4] Schwartz GG Steg PG Szarek M Bhatt DL Bittner VA Diaz R Alirocumab and cardiovascular outcomes after acute coronary syndrome. N Engl J Med. (2018) 379(22):2097–107. 10.1056/NEJMoa1801174 30403574

[5] Sabatine MS Giugliano RP Keech AC Honarpour N Wiviott SD Murphy SA Evolocumab and clinical outcomes in patients with cardiovascular disease. N Engl J Med. (2017) 376(18):1713–22. 10.1056/NEJMoa1615664 28304224

[6] Karatasakis A Danek BA Karacsonyi J Rangan BV Roesle MK Knickelbine T Effect of PCSK9 inhibitors on clinical outcomes in patients with hypercholesterolemia: a meta-analysis of 35 randomized controlled trials. J Am Heart Assoc. (2017) 6(12):e006910. 10.1161/JAHA.117.006910 29223954 PMC5779013

[7] Biccirè Flavio G Häner J Losdat S Ueki Y Shibutani H Otsuka T Concomitant coronary atheroma regression and stabilization in response to lipid-lowering therapy. JACC. (2023) 82(18):1737–47. 10.1016/j.jacc.2023.08.019 37640248

[8] Räber L Ueki Y Otsuka T Losdat S Häner JD Lonborg J Effect of alirocumab added to high-intensity statin therapy on coronary atherosclerosis in patients with acute myocardial infarction: the PACMAN-AMI randomized clinical trial. JAMA. (2022) 327(18):1771–81. 10.1001/jama.2022.5218 35368058 PMC8978048

[9] Nicholls SJ Kataoka Y Nissen SE Prati F Windecker S Puri R Effect of evolocumab on coronary plaque phenotype and burden in statin-treated patients following myocardial infarction. JACC: Cardiovascular Imaging. (2022) 15(7):1308–21. 10.1016/j.jcmg.2022.03.002 35431172

[10] Shin D Kim S Lee H Lee HC Lee J Park HW PCSK9 Stimulates syk, PKC*δ*, and NF-*κ*B, leading to atherosclerosis progression independently of LDL receptor. Nat Commun. (2024) 15(1):2789. 10.1038/s41467-024-46336-2 38555386 PMC10981688

[11] Basiak M Kosowski M Hachula M Okopien B. Impact of PCSK9 inhibition on proinflammatory cytokines and matrix metalloproteinases release in patients with mixed hyperlipidemia and vulnerable atherosclerotic plaque. Pharmaceuticals (Basel). (2022) 15(7):802. 10.3390/ph15070802 35890100 PMC9324132

[12] Huang L Li Y Cheng Z Lv Z Luo S Xia Y. PCSK9 Promotes endothelial dysfunction during sepsis via the TLR4/MyD88/NF-*κ*B and NLRP3 pathways. Inflammation. (2023) 46(1):115–28. 10.1007/s10753-022-01715-z 35930089

[13] Lupo MG Marchianò S Adorni MP Zimetti F Ruscica M Greco MF PCSK9 Induces rat smooth muscle cell proliferation and counteracts the pleiotropic effects of simvastatin. Int J Mol Sci. (2021) 22(8):4114. 10.3390/ijms22084114 33923431 PMC8073479

[14] Zhang Q Miao M Cao S Liu D Cao Z Bai X PCSK9 Promotes vascular neointimal hyperplasia through non-lipid regulation of vascular smooth muscle cell proliferation, migration, and autophagy. Biochem Biophys Res Commun. (2025) 742:151081. 10.1016/j.bbrc.2024.151081 39632291

[15] Liu S Wu J Stolarz A Zhang H Boerma M Byrum SD PCSK9 Attenuates efferocytosis in endothelial cells and promotes vascular aging. Theranostics. (2023) 13(9):2914–29. 10.7150/thno.83914 37284459 PMC10240829

[16] Schuster S Rubil S Endres M Princen HMG Boeckel JN Winter K Anti-PCSK9 antibodies inhibit pro-atherogenic mechanisms in APOE*3Leiden.CETP mice. Sci Rep. (2019) 9(1):11079. 10.1038/s41598-019-47242-0 31366894 PMC6668462

[17] Momtazi-Borojeni AA Sabouri-Rad S Gotto AM Pirro M Banach M Awan Z PCSK9 And inflammation: a review of experimental and clinical evidence. Eur Heart J Cardiovasc Pharmacother. (2019) 5(4):237–45. 10.1093/ehjcvp/pvz022 31236571

[18] Yurtseven E Ural D Baysal K Tokgözoğlu L. An update on the role of PCSK9 in atherosclerosis. J Atheroscler Thromb. (2020) 27(9):909–18. 10.5551/jat.55400 32713931 PMC7508721

[19] Stoekenbroek RM Lambert G Cariou B Hovingh GK. Inhibiting PCSK9 - biology beyond LDL control. Nat Rev Endocrinol. (2018) 15(1):52–62. 10.1038/s41574-018-0110-5 30367179

[20] Matter MA Paneni F Libby P Frantz S Stähli BE Templin C Inflammation in acute myocardial infarction: the good, the bad and the ugly. Eur Heart J. (2023) 45(2):89–103. 10.1093/eurheartj/ehad486 PMC10771378 37587550

[21] Tucker B Vaidya K Cochran BJ Patel S. Inflammation during percutaneous coronary intervention-prognostic value, mechanisms and therapeutic targets. Cells. (2021) 10(6):1391. 10.3390/cells10061391 34199975 PMC8230292

[22] Borhani S Hassanajili S Ahmadi Tafti SH Rabbani S. Cardiovascular stents: overview, evolution, and next generation. Prog Biomater. (2018) 7(3):175–205. 10.1007/s40204-018-0097-y 30203125 PMC6173682

[23] Grewe PH Deneke T Machraoui A Barmeyer J Müller KM. Acute and chronic tissue response to coronary stent implantation: pathologic findings in human specimen. J Am Coll Cardiol. (2000) 35(1):157–63. 10.1016/S0735-1097(99)00486-6 10636274

[24] Madder RD Kubo T Ino Y Kameyama T Terada K VanOosterhout S Target lesion lipid content detected by near-infrared spectroscopy after stenting and the risk of subsequent target lesion failure. Arterioscler Thromb Vasc Biol. (2021) 41(7):2181–9. 10.1161/ATVBAHA.120.315617 33980034

[25] Kim W Kook H Park S Heo R Park J Shin J Impact of post-PCI lipid core burden Index on angiographic and clinical outcomes: insights from NIRS-IVUS. Circ Cardiovasc Imaging. (2025) 18(6):e017740. 10.1161/CIRCIMAGING.124.017740 40340593

